# Treatment-Free Remission in Chronic Myeloid Leukemia and New Approaches by Targeting Leukemia Stem Cells

**DOI:** 10.3389/fonc.2021.769730

**Published:** 2021-10-28

**Authors:** Yilin Chen, Jing Zou, Fanjun Cheng, Weiming Li

**Affiliations:** Department of Hematology, Union Hospital, Tongji Medical College, Huazhong University of Science and Technology, Wuhan, China

**Keywords:** chronic myeloid leukemia, tyrosine kinase inhibitors, leukemia stem cells, discontinuation, treatment-free remission

## Abstract

The therapeutic landscape for chronic myeloid leukemia (CML) has improved significantly with the approval of tyrosine kinase inhibitors (TKIs) for therapeutic use. Most patients with optimal responses to TKIs can have a normal life expectancy. Treatment-free remission (TFR) after discontinuing TKI has increasingly become a new goal for CML treatment. However, TKI only “control“ CML, and relapse after discontinuation has become a key factor hindering patient access to attempt TFR. In this study, we reviewed studies on TKI discontinuation, including both first and second-generation TKI. We also reviewed predictors of relapse, new monitoring methods, and strategies targeting leukemic stem cells.

## Introduction

Chronic myeloid leukemia (CML) is a clonal myeloproliferative disease characterized by the BCR-ABL fusion gene as a result of t (9;22) (q34;q11) translocation (1). Tyrosine kinase inhibitors (TKIs) have revolutionized the treatment of CML and significantly improved the outcomes for CML patients. Currently, several TKIs are approved for the treatment of CML: imatinib, dasatinib, nilotinib, bosutinib, ponatinib (2–6). Most CML patients who benefit from TKIs have a life expectancy similar to that of the general population (7, 8). However, the adverse events and TKI costs associated with lifelong treatment considerably affect affected patients’ quality of life and dependence on TKI, ultimately affecting treatment response (9–11). An attempt at treatment discontinuation can be considered in some patients to mitigate the adverse events or reduce treatment costs. In addition, female patients of childbearing potential can benefit from stoppi ng TKI to lessen the risk of teratogenic effects (12). Results from multiple trials suggest that patients with a stable deep molecular response (DMR) can safely discontinue TKI without relapse, but close monitoring is recommended (13, 14). Treatment-free remission (TFR) is increasingly becoming a therapeutic goal for patients with DMR (15, 16). However, more studies analyzing predictive factors, new monitoring methods, new strategies targeting stem cells are required to improve current TFR rates. The purpose of this review is to provide updated data on TKI discontinuation studies and analyze predictive factors, monitoring methods, and novel strategies to improve TFR.

## Outcomes With First- and Second-Generation TKI Discontinuation

The pioneering TFR trials with imatinib demonstrated the feasibility of TKI discontinuation. With the advent of second-generation TKI discontinuation trials, data regarding TFR are increasingly available. As clinical trials of TFR discontinuation show a favorable TFR rate, many patients in the real world can attempt to stop TKI to achieve TFR. Here, we mainly present the data on discontinuation imatinib, dasatinib, and nilotinib discontinuation in clinical trials and the real world (**Tables 1** and **2**). In addition, we discuss the feasibility of the second TKI discontinuation attempt after an unsuccessful attempt.

**Table 1:** Studies of Imatinib Discontinuation.

Study	Eligibility criteria	No. Pts	Relapse definition	TFR rate	Median time to relapse	Patients regaining MR after reinitiating TKIs
STIM (13, 17)	UMRD ≥ 2 years; Imatinib treatment ≥3 years	100	Two successive increased BCR-ABL transcripts	41% at 12 months 38% at 24 months 38% at 60 months	2.5 months (range, 0.9 to 22.3)	55/57 regained UMRD with a median of 4 months
A-STIM (18)	UMRD ≥ 2 years; Imatinib treatment ≥3 years	80	Loss of MMR	64% at 12 and 24 months 61% at 36 months	4 months (range, 2 to 17)	31/31 regained MMR
KID (19)	UMRD >2 years; Imatinib treatment ≥3 years	90	Loss of MMR	62.2% at 12 months 58.5% at 24 months	3.3months (range, 0.9 to 20.8)	37/37 regained MMR at a median of 3.9 months (range, 0.5 to 11.1)
ISAV (20)	UMRD ≥ 1.5 years; Imatinib treatment ≥2 years	107	Loss of MMR	52.6% at 5 years	5 months (range, 1.4 to 55.6)	95.9% regained MMR at a median of 1.9 months
TWISTE (21)	UMRD ≥ 2 years; Imatinib treatment ≥3 years	40	Loss of MMR	47.1% at 2 years 45% at 8 years	15/22 relapsed within 6 months	22/22 regained UMRD at a median of 3.3 months (range, 0.0 to 17.4)
JALSG-STIM2 (13, 22)	MR4.0≥2 years; Imatinib treatment ≥3 years	68	Loss of MMR	67.6% at 12 months	22/25 relapsed within 12 months	25/25 regained MMR within 6 months
DOME-STIC (23)	MR4.0 ≥ 2 years	99	Loss of MR4.0	69.6% at 6 months 68.6% at 12 months 64.3% at 24 months	NR	25/26 regained MR4.0 within 12 months
TRAD (24)	MR4.5≥ 2 years; Imatinib treatment ≥3 years	131	Loss of MMR and/or MR4.0	69.9% at 12 months	53/58 relapsed within 6 months	49/54 regained MR4.5 at a median of 2.48 months

UMRD, undetectable minimal residual disease; MMR, major molecular response; MR4.0, molecular response of 4‐log reduction of BCR‐ABL1 transcripts; MR4.5, molecular response of 4.5‐log reduction of BCR‐ABL1 transcripts; TFR, treatment‐free remission; MR, molecular response

**Table 2:** Studies of dasatinib and nilotinib discontinuation.

Study	Eligibility criteria	No. Pts	Treatment	Relapse definition	TFR rate	Median time to relapse	Patients regaining MR after reinitiating TKIs
DADI (25)	MR4.0 ≥1 year on dasatinib	63	First-line or subsequent line dasatinib	Loss of MR4.0	44% at 36 months	33/33 relapsed within 7 months	33/33 regained MR4.0 within 6 months
D-STOP (26)	MR4.0 ≥ 2 years on dasatinib	54	First-line or subsequent line dasatinib	Two successive BCR-ABL1-positive	62.9% at 12 months; 59.3% at 24 months	20/22 relapsed within 7 months	22/22 regained MR4.0 within 12 months
DASFREE (27)	MR4.5 ≥ 1year on dasatinib	84	First-line or subsequent line dasatinib	Loss of MMR	48% at 1 year; 46% at 2 years	NR	44/45 regained MMR at a median of 2 months (range, 1 to 4) 43/45 regained MR4.5 at a median of 3 months (range, 2 to18)
First-line DADI (28)	MR4.0 or deeper≥1 year, dasatinib treatment ≥2 years	58	First-line dasatinib	Loss of MR4.0	55.2% at 6 months	2 months (range, 1.3 to 2.7)	23/25 regained MR4.0 within 12 months
ENEST Freedom (29–31)	MR4.5 ≥ 2 years; nilotinib ≥3 years	190	First-line nilotinib	Loss of MMR	51.6% at 48 weeks; 48.9% at 96 weeks 42.6% at 5 years	88/94 relapsed within 48 weeks	90/91 regained MMR(91.2% within 12 weeks) 84/91 regained MR4.5
STAT2 (32)	MR4.5≥ 2 years on nilotinib	78	Second-line nilotinib	Loss of MMR	67.9% at 1 year; 62.8% at 2 years	3.4 months (range, 1.8 to 5.8)	25/29 regained MR4.5 (50% within 3.5months)
NILSt (33)	MR4.5 at 2 years on nilotinib	112	First-line and second-line nilotinib	Loss of MR4.5	60.9% at 1 year; 60.9% at 3 years	4 months (range, 2 to 11)	33/33 regained MR4.5 at a median of 2.8 months (range, 1 to 17)
ENESTop (34–36)	MR4.5 ≥2 years on nilotinib	126	Second-line nilotinib	Loss of MMR or confirmed loss of MR4.0	58% at 48 weeks; 53% at 96 weeks; 46.0% at 192 weeks; 42.9% at 5 years	49/53 relapsed within the 24 weeks	58/59 regained MMR; 56/59 regained MR4.0; 55/59 regained MR4.5 at a median of 2.9 months (range, 0.9-22.5)
STOP 2G- TKI (14)	MR4.5≥ 2 years; TKI treatment ≥3 years	100	Dasatinib or nilotinib	Loss of MMR	63.33% at 1 year 53.57% at 4 years	4 months (range, 1 to 38)	25/25 regained MMR at a median of 2 months (range, 1 to 6)
LAST (37)	MR4.0 ≥2 years; TKI treatment ≥3 years	172	Imatinib, dasatinib, nilotinib, or bosutinib	Loss of MMR	60.8% at 36 months	4 months (range, 1.5 to 41.3)	55/59 regained MR4.0
RU-SKI (38)	MR4.0 ≥ 2 years; TKI treatment ≥3 years	98	Imatinib, dasatinib, or nilotinib	Loss of MMR	53% at 12 months 52% at 24 months	3 months (range, 1 to 28)	47/47 regained MMR at a median of 3 months (range, 0.4 to 15)
Gabriel Etienne (39)	MR4.5 ≥ 2 year	95	Imatinib, 2-3G TKI	Loss of MMR	55.1% at 12 months; 46.9% at 48 months	NR	NR
Argentina Stop Trial (40)	MR4.0 ≥ 2 years; TKI treatment ≥3 years	46	Imatinib, dasatinib, nilotinib	Loss of MMR	80.2% at 6 months	NR	12/15 regained MMR at a median of 3 months (range, 1 to 8); 9/15 regained MR4.0 at a median time of 3 months (range, 1 to 5)
EURO-SKI (41)	MR4.0 ≥1 year; TKI treatment ≥3 years	758	Imatinib, dasatinib, or nilotinib	Loss of MMR	61% at 6 months 50% at 24 months 49% at 36 months	297/373 relapsed within 6 months	321/373 regained MMR at a median of 2.8 months; 302/373 regained MR4.0 at a median of 3.7 months

MMR, major molecular response; MR4.0, molecular response of 4‐log reduction of BCR‐ABL1 transcripts; MR4.5, molecular response of 4.5‐log reduction of BCR‐ABL1 transcripts; TFR, treatment‐free remission; MR, molecular response.

### Imatinib Discontinuation

In the prospective, multicenter Stop Imatinib (STIM) trial, 100 patients with undetectable minimal residual disease (UMRD) for more than two years with imatinib were enrolled. Molecular relapse was defined as a significant increase in BCR-ABL transcripts during two consecutive assessments. The estimated molecular relapse-free survival (MRFS) was 41% and 38% at 12 months and 24 months (17). In the Long-Term Follow-Up of the STIM1 study, the MRFS rate was 38% at 60 months. 80% of patients displayed relapsed in the first 6 months. 96.5% (55/57) patients achieved a second UMRD with a median of 4 months of retreatment (13). With longer follow‐up intervals, it became clear that low levels of transcripts were detectable in some patients and did not increase further. In some studies, molecular recurrence was defined as the loss of MMR. The A-STIM study estimated the TFR rates as 64% at 12 and 24 months and 61% at 36 months (18). The KID study reported similar TFR rates of 62.5% and 58.5%, at 12 and 24 months as in the A-STIM study (19). In studies with longer follow-ups, the 5-year MRFS rate in the ISAV study was 47.4%, and the 8-year MRFS rate in the TWISTER study was 45% (20, 21). These results suggest that some patients can achieve a long-term safe and stable response after discontinuation of imatinib. Due to difficulty achieving UMRD in many laboratories, DMR (MR4.0 or MR4.5) for two years has increasingly become an inclusion criterion in subsequent studies, JALSG-STIM213, DOMESTIC, and the TRAD study, which reported similar 12-month TFR rates of 67.6%, 68.6%, and 69.9%, respectively (22–24).

Molecular relapse commonly occurred in the first six months after imatinib withdrawal (13, 18, 24). In the TWISTER study with long-term follow-up, the latest relapse was detected 27 months after stopping imatinib (21). A recent study showed the residual rate of molecular recurrence after two years of discontinuing imatinib was estimated to be 18% (42). Even though some patients with a stable DMR or deeper molecular response can achieve TFR after imatinib discontinuation, long-term molecular follow-up remains mandatory for CML patients in TFR.

### Dasatinib Discontinuation

The DADI trial was a prospective multicenter trial investigating the safety and efficacy of discontinuing first-line or subsequent dasatinib (25). 63 patients taking dasatinib and with confirmed stable DMR (MR4.0) for at least one year tried to stop dasatinib. The estimated overall TFR was 44% at 36 months. Most patients experienced molecular relapses within the first seven months after discontinuation. All relapsed patients achieved molecular response after retreatment with dasatinib. The presence of imatinib resistance was a significant risk factor for molecular relapse. In the D-STOP trial, 54 patients with steady DMR for two years had discontinued dasatinib. The estimated treatment-free survival at 12-month and 24 months were 62.9% and 59.3%, respectively (26). Moreover, in the DASFREE study, 84 patients discontinued first and second-line of dasatinib therapy; 48% and 46% of all patients were found with maintained TFR at one and two years. Patients with the first and second-line treatment showed comparable TFR rates at two years, 51%, and 42%, respectively. The TFR rate was the same for patients resistant to prior TKI and intolerant to prior TKI, both at 44% (27). The first-line DADI trial assessed the safety and efficacy of discontinuing first-line dasatinib; 58 patients with DMR for one year discontinued dasatinib after receiving first-line dasatinib for at least 24 months. The TFR rate at 6 months was 55.2% (28). These findings indicate that discontinuation of first-or subsequent-line dasatinib after a sustained DMR was feasible.

### Nilotinib Discontinuation

The ENESTfreedom study was the first single-arm, phase 2 clinical trial to evaluate the possibility of stopping first-line nilotinib. 190 patients with at least two years of DMR (MR4.5) of frontline nilotinib therapy were enrolled. Patients restarted nilotinib following the loss of MMR. After stopping nilotinib, 98 (51.6%), and 93 patients (48.9%) remained in MMR at 48 and 96 weeks, respectively (29, 30). At the 5-year data cut-off, 81/190 patients (42.6%) were still in sustained TFR, while 90/91 patients (98.9%) regained MMR after restarting nilotinib treatment (31). Several clinical studies have evaluated the possibility of stopping second-line nilotinib. In the STAT2 trial, 78 patients with stable DMR (at least MR4.5) after 2-year consolidation therapy with nilotinib attempted to discontinue nilotinib. The TFR rates at 12 and 24 months were 67.9% and 62.8%, respectively (32). Similarly, patients who achieved MR4.5 after first-line imatinib or nilotinib therapy were given nilotinib consolidation for up to 24 months in the NILSt trial, and 87 of these patients proceeded to discontinuation of nilotinib. The TFR rate at 1 and 3 years both was 60.9% after nilotinib discontinuation (33). In the ENESTop study, 126 patients with sustained MR4.5 after second-line nilotinib treatment entered the TFR phase. The TFR rates at 48, 96, and 192 weeks were 58%, 53%, and 46.0%, respectively. Most patients requiring nilotinib retreatment rapidly regained MMR, MR4.0, or MR4.5 (34, 35). The long-term follow-up results showed a 5-year TFR rate of 42.9% (36), comparable to the results of the ENESTfreedom study (31). These results illustrate the long-term durability of TFR in patients with first or second-line nilotinib. In addition, the subgroup analysis of the ENESTop study showed that patients switching to nilotinib due to intolerance, resistance, and physician preference had a similar TFR rate at 48 weeks: 30 of 51 (58.8%), 16 of 30 (53.3%), and 27 of 44 (61.4%), respectively (43). Accordingly, first-line resistant or intolerant patients who discontinue second-line TKI still have the chance to achieve TFR.

In the ENSTnd study, with ≥10 years follow-up in newly diagnosed CML patients, the estimated cumulative rates of TFR eligibility [estimated using ENESTfreedom criteria (31)] with nilotinib 300-mg twice-daily, nilotinib 400-mg twice-daily, and imatinib, respectively, at 5 years were 20.9%, 20.6%, and 11.0% and 10 years were 48.6%, 47.3%, and 29.7% (44). The JALSG CML212 study compared the achievement of MR4.5 in newly diagnosed CML patients between nilotinib and dasatinib (45). The MR4.5 rates by 12, 24, and 36 months were 25.6%, 37.4%, and 40.5% in the nilotinib arm and 23.4%, 36.6%, and 44.5% in the dasatinib arm, respectively, with no significant difference, indicating nilotinib and dasatinib were equally effective for CML-CP patients in achieving MR4.5. Among patients reaching TFR eligibility, approximately 50% maintained their responses after TKI discontinuation, and dasatinib, nilotinib TKIs did not increase the overall TFR success rate (**Tables 1**, **2**). Therefore, it can be speculated that only 5% and 10% of newly diagnosed CML patients achieved TFR at 5 years of treatment with imatinib, nilotinib (or dasatinib), compared with 15% and 24% at 10 years of treatment.

### Multiple TKIs

The STOP 2G- TKI study estimated the safety of second-generation TKI discontinuation in CML patients receiving dasatinib or nilotinib (14). All patients included were treated for at least three years and had two years of stable MR4.5. TFR rates at 12 and 48 months were 63.33% and 53.57%, respectively. The LAST study presented a TFR rate of 60.8% at 36 months. The median time to molecular recurrence was the same as that in STOP -2G TKI, both 4 months (37). In the RU-SKI study, 98 patients treated with first or second-generation TKI were included. Survival without MMR loss at 12 and 24 months after TKI discontinuation was 53% and 52%, respectively (38). An observational study by Gabriel Etienne et al. exhibited MRFS rates of 51.8% and 43.8% at 12 and 60 months, respectively, similar to the RU-SKI study (39). Furthermore, the Argentina Stop Trial (AST) trial recently included 46 patients also showed a high MRFS at 6 months of 80.2% (40). Evidence from these trials demonstrates that patients with DMR can achieve favorable MRFS. The EURO-SKI study enrolled the largest number of patients (n=758) treated with imatinib, nilotinib, or dasatinib who achieved confirmed DMR for at least one year. The MRFS rate of these patients was 61%, 50%, 49% at 6, 24 months, and 49% at 36 months (41). With 72 months follow-up, 12 out of 111 patients (10.8%) who were in TFR at 36 months, subsequently lost MMR. Interestingly, 1% (1/98) of patients at MR4.0 at 36 months relapsed, yet the risk of relapse for those not at MR4.0 was 85% (11/13), indicating the molecular response at 36 months after TKI discontinuation was highly predictive of molecular relapse (46). The results suggested that the frequency of continued molecular monitoring after three years may depend on the molecular status. The ELN recommendations suggest continuous measurement of BCR-ABL1 levels every 3 months (16). BCR-ABL1 levels should be monitored every 3 months for patients not in MR4.0, while those in MR4.0 may only need an evaluation of BCR-ABL1 levels once or twice a year.

### Second Attempt to TKI Discontinuation

An increasing body of evidence suggests that approximately one-half of CML patients with steady DMR can successfully discontinue TKI, and patients with molecular relapse can regain DMR rapidly after restarting TKI (13, 27, 31). Several studies explored the feasibility of the second discontinuation on patients with molecular relapse after the first attempt of TKI discontinuation. In the TRAD study, 32 patients with MR4.5 for 12 months with dasatinib treatment after failed imatinib discontinuation attempted to achieve a second TFR. After dasatinib discontinuation, the estimated TFR rate was 20.4% at 6 months (24). In the KID study, 15/23 patients (62.1%) who attempted second imatinib discontinuation experienced molecular relapse after a median of 2.9 months (47). In the RE-STIM study, 70 patients reattempted TKI discontinuation after a first unsuccessful attempt. The TFR rates at 12, 24, and 36 months were 48%, 42%, and 35%, respectively. Patients who lost MR4.5 later than the median time (>3 months) after the first TKI discontinuation experienced a remarkably lower rate of molecular relapses after the second attempt than others (48). In the enlarged RE-STIM study, 106 patients were enrolled with a median follow-up of 41 months after the second discontinuation. The TFR rates were 44.3%, 38.5%, and 33.2% at 24, 36, and 48 months after the second TKI discontinuation. The speed of molecular relapse after the first TKI discontinuation remained significantly associated with the second TFR (49). Those results showed that some patients who failed the first discontinuation could safely and successfully discontinue TKIs a second time, especially for patients who relapsed later than the median time during a first discontinuation attempt. Further investigations on the predictors that can identify patients for second TKI discontinuation and new strategies to improve the TFR rate are required.

### Real‐World TKI Discontinuation

In recent years, several studies have reported the outcome of TKI withdrawal in patients with CML outside clinical trials. A study from a Spanish research group described the outcomes of 236 patients after TKI discontinuation in clinical practice. The TFR rate at 4 years was 64%. TKI treatment duration less than 5 years and MR4.5 duration shorter than 4 years were both associated with a higher incidence of molecular recurrence (50). Another observational study by an Italian research group reported an estimated TFR at 12 months of 69% for patients discontinuing first- and second-line TKI, including 68% for those with imatinib, and 73% for those with second-generation TKI (51). A Swedish group reported that 62.2% of patients discontinuing TKI in clinical practice remained in TFR at the last follow-up (median follow-up time 1.6 years), consistent with the Spanish and Italian studies (52). A single-institution retrospective study also showed that 65% (65/100) patients maintained MR4.5 after a median follow-up of 30 months after discontinuation of TKI. MR4.5 duration for at least six years before discontinuation has been associated with a considerably low risk of loss of MR4.5 (53). A retrospective study assessed first, second, and third attempts to stop TKIs in patients in the real world (54); 28 out of a total of 53 patients (53.4%) achieved sustained TFR after the first attempt; subsequently, 4 of the 10 patients (37.5%) who attempted the second discontinuation successfully achieved TFR. Patients with molecular relapse achieved MMR soon after restarting TKI. All six experienced a loss of MR4.5 after the third attempt to stop TKI. Consistent with the RE-STIM study, loss of MR4.5 at 3 months was an important predictive factor for achievement of second TFR. The above studies suggest that TKI discontinuation in CML is common and feasible outside of clinical trials. Importantly, second successful TKI discontinuation can still be achieved in appropriately selected patients in clinical practice.

## De-Escalation TKI Dose – An Alternative Strategy

Several clinical studies have evaluated whether TKI dose reductions can maintain molecular responses. In a study assessing whether high-dose imatinib could be safely reduced to a standard dose without increasing the risk of losing DMR, MMR was maintained in 90% of 68 patients (61/68) with 400 mg imatinib (55). In the NILO-RED study, 67 patients with MMR or deeper molecular response switched from a standard to low dose Nilotinib. The 12-month probability of survival without MMR loss was 97% (56). The non-randomized DESTINY trial assessed the efficacy of a novel approach characterized by halving the dose of a TKI for 12 months and subsequently discontinuing the TKI completely for a further 24 months (57). 174 patients who received TKI for at least 3 years and achieved MMR at least for 12 months were included, of which 125 patients were in the MR4.0 cohort and 49 patients were in the MMR cohort. During the 12 months of half-dose therapy, 2% of patients with MR4.0 and 19% with MMR experienced molecular recurrence. All relapsing patients rapidly regained MMR or better within 4 months of resumption of full-dose TKI. These results indicated the feasibility of lower TKI doses in maintaining responses in patients with stable responses. Fassoni et al. applied a simple mathematical model that describes the time course of TKI response in CML as a dynamic process (58). Their findings showed that dose reduction retained the long-term efficacy in patients who achieved stable molecular remission.

Furthermore, long-term follow-up of the DESTINY trial presented 2-year MRFS rates of 72% and 36% for the MR4.0 and MMR groups after TKI discontinuation (59). Relapse-free survival in the DESTINY trial appeared to be better than in the EURO-SKI study, with 24-month relapse-free rates of 50% (41). Some studies had similar results as the DESTINY trial, with the reduced dose group having a higher TFR rate than the full-dose group. A retrospective study compared the efficacy of TKI discontinuation in patients with reduced and standard doses. Higher TFR rates were observed in the low-dose group at 12 (80% vs.56.8%, *P*=0.03) and 60 months (58.8% vs. 47.5%, *P*=0.14) (60). Another recent study evaluated the efficacy and safety of TKI dose reduction in 246 patients who reached MMR due to TKI intolerance in the real world. The 3-year MRFS in MMR and MR4.0 were 94.1% and 87.1%. Interestingly, of the 94 patients in MMR, 54.2% achieved a molecular response level of MR4.0 or higher with a lowered dose. 76 patients discontinued TKI after 1.76 years of low dose treatment, and the 2-year TFR rate in these patients was 74.1% (61). Consistent with the DESTINY trial, a TKI step-down strategy before treatment discontinuation may be a promising strategy to improve TFR.

A possible mechanism underlying the improved TFR and response in the reduced group may be altered immune response against leukemia induced by TKI de-escalation (62). In a recent study, an ordinary differential equation model including an antileukemic immunologic effect was used to assess the predictive impact of different immunological configurations on TKI discontinuation. Dose optimization could be considered for class C patients since they could achieve TFR only if an optimal balance between leukemia abundance and immunologic activation were achieved before treatment cessation (63). In the updated DESTINY study, BCR-ABL1IS values monitored during dose reduction were strongly associated with individual relapse risk after TKI discontinuation. It was recommended that after a 12-month dose reduction period, patients should only stop treatment if they are below MMR and have a negative/low BCR-ABL1 slope; patients with a high slope should return to full-dose TKI therapy because of the high likelihood of relapse (64). The changes in BCR-ABL1 kinetics changes during dose reduction may help clinicians decide whether to stop TKI treatment after the dose reduction period.

## Predictive Factors of Molecular Recurrence

Identifying predictive factors of molecular recurrence contributes to the clinical prediction of a successful attempt and the ability to sustain TFR. Here, we discuss the clinical, immunological, and other factors that may help identify patients suitable for TKI discontinuation.

### Clinical Indicators

During the Stop Imatinib (STIM1) study, patients with low or intermediate Sokal risk scores and IM duration longer than 54 months experienced a considerably lower rate of molecular recurrence (13). The TKI treatment duration was also associated with a favorable prognosis in other studies (23, 27, 43, 50). The EURO-SKI trial documented that longer DMR durations were associated with an increased probability of TFR (41). The recent TRAD study further reported that one additional year of MR4.0 duration decreased the risk of TFR failure by 14.0% and proposed six years as the shortest imatinib duration (65). Several studies reported the depth of molecular response at the study baseline could predict TFR (22, 25, 32). In this regard, the ENESTfreedom study described patients maintaining MR4.5 at 48 weeks experienced a better TFR rate at 5 years than patients lacking MR4.5 (86.0% for patients in MR4.5 vs. 33.3% for patients in MR4.0 and 20.0% for patients in MMR), suggesting a stable MR4.5 may be a potential predictor of successful TFR (31). In the DADI trial, imatinib resistance was a significant risk factor for molecular relapse (25). Nevertheless, patients switching to nilotinib due to imatinib intolerance and resistance in the ENESTop study interestingly experienced a similar TFR rate at 48 weeks (58.8%vs. 53.3%) (43). Furthermore, in the multiple, TKIs discontinuation study, first-line 2-3G TKIs compared to imatinib were significant predictors of MRFS (39). Imatinib withdrawal syndrome was associated with a higher probability of sustained MMR than patients without withdrawal syndrome (79.5% vs. 49.2%, *P*=0.003) in the KID study (19). However, no association between withdrawal syndrome development and the rate of molecular relapses was found in the RU-SKI study (38). These results substantiate patients with deeper molecular responses and a longer duration of molecular responses before stopping TKI had higher rates of TFR.

Several studies reported that patients with e14a2 transcripts had a higher probability of maintaining TFR than those with e13a2 transcripts (66, 67). In a recent study, BCR-ABL1 levels at the first month after TKI discontinuation were associated with successful TFR. Moreover, the slopes obtained with the values at baseline at 1 and 2 months were also significantly different between patients with and without sustained MMR (68). In another study, the time required to halve the initial BCR-ABL1 transcripts value was the strongest independent predictor of sustained TFR. Patients with halving times less than 9.35 days had a significantly higher TFR rate than those with halving times greater than 21.85 days (80% vs.4%, *P*<0.01) (69). Digital PCR (dPCR) is a technique that promises to achieve highly accurate absolute nucleic acid quantification with higher precision and improved daily reproducibility compared to real-time PCR (RT-PCR) (70). In the ISAV study, age and dPCR results were significant predictors of molecular recurrence (20). Those results corroborate the critical importance of the BCR-ABL1 transcript type and the kinetics of BCR-ABL1 decline on long-term outcomes. In addition, BCR-ABL1 levels monitored by dPCR, if available at TKI discontinuation, should be taken into account in selecting patients suitable for discontinuation.

### Immunological Indicators

After discontinuation of TKI therapy, relapse depends significantly on an individual’s leukemia-specific immune response (63). Immunological indicators are also emerging as predictive biomarkers of molecular relapse. In the DADI trial, high NK-cell counts, including CD3– CD56+ and CD16+ CD56+ cells, and high counts of NK-cell large granular lymphocytes (CD57+ 56+) were significantly associated with TFR (25). During the three years follow-up of the D-STOP study, CD3- CD56+ NK, CD16+ CD56+ NK, and CD57+ CD56+ NK large granular lymphocyte (NK-LGL), CD8+ CD4- cytotoxic T cell, and CD57+ CD3+ T-LGL cell numbers in patients with TFR were transiently elevated after 12 months but returned to basal levels after 24-month dasatinib consolidation. Silent responses of the T/NK subsets to dasatinib throughout consolidation therapy were significant for maintaining TFR (26). In an immunological study within the EURO-SKI study, the MRFS rate was higher in patients with a higher relative proportion of NK cells than the median, compared with patients with lower NK-cell proportion (73% vs. 51% at 6 months, *P*=0.02). Moreover, patients with higher than median CD56^bright^ NK cells exhibited a decreased MRFS at 6 months (52% vs 70%, *P*=0.14) (71). In the IMMUNOSTIM study, the CD56^dim^ NK cell count was an independent prognostic factor of TFR (72). It is widely acknowledged that NK cell functions are under the control of surface inhibitory and activating receptors, such as Natural killer group 2(NKG2) and killer immunoglobulin-like receptors (KIR). Patients homozygous for KIR A haplotype experienced an increased cumulative TFR (73). In another study, the KIR2DL5B-positive genotype was independently related to a delayed second DMR after TKI restart (74). KIR2DS3 was also showed to be more frequent in patients who relapsed after TKI discontinuation (75). HLA polymorphisms have been observed to be associated with TFR (76). Natural killer group 2D receptor (NKG2D) is an activating receptor expressed on NK cells. NKG2D HNK1/HNK1 (high-cytotoxic activity-related allele on NKG2D hb-1) haplotype has been associated with the faster acquisition of MR4.5 (77). NKG2A downregulation by dasatinib enhanced NK cell cytotoxicity and accelerated molecular responses (78). These results demonstrated that NKG2D gene polymorphisms and NKG2A might serve as biomarkers for predicting TFR following dasatinib treatment.

Intriguingly, in the EURO-SKI trial, increased level of CD86 receptor, the ligand of CTLA-4 on plasmacytoid dendritic cells was associated with CD8+ CTLs exhaustion and higher risk of relapse after TKI cessation (79). Patients with CXorf48-specific CTL-negative displayed an increased relapse rate compared to patients with CXorf48-specific CTL-positive (63.6% vs. 0%), indicating CXorf48 could be a promising therapeutic target of Leukemic stem cells (LSCs) to achieve TFR (80). In a recent study, a notable increase in unconventional CD8± T cells expressing TCRγβ+ was observed in patients with TFR (75). Monocytic myeloid-derived suppressor cells (Mo-MDSCs) were concomitantly decreased in patients who achieved TFR (81). Such evidence showed that both immune suppressors and effectors in immunobiology contribute to underlying successful TFR.

### Other Indicators

Several studies have explored the effect of genetic factors on the successful discontinuation of TKI. Downregulation of Plasma miR-215 and, microRNA-148b has been associated with successful discontinuation of imatinib (82, 83). The results from whole-exome sequencing revealed variants in genes CYP1B1, ALPK2, and IRF1 in patients with relapse and one variant in gene PARP9 in patients without relapse (84). In a EURO-SKI sub-trial, patients with high transcript levels of the ABCG2 efflux transporter underwent a higher risk of relapse (85).

## The New Technology of Molecular Monitoring

New monitoring technology may contribute to the identification of patients who are eligible to discontinue TKI in the future. In recent years, digital PCR (dPCR) has emerged as one of the most promising tools. Some recent findings indicated that dPCR is more efficient than RQ-PCR for monitoring MRD in CML and contributes to selecting patients more compatible with TFR (37, 86–88). In the study by Nicolini et al., low levels of BCR-ABL1 were assessed by dPCR in 175 patients at the time of discontinuation of imatinib. The duration of TKI (≥74.8 months) and dPCR (≥ 0.0023%(IS)) were the two identified predictive factors of molecular recurrence (86). Colafigli, G et al. have reported that dPCR-positive patients presented significantly increased risks of molecular recurrence compared to dPCR-negative patients (50%vs.14%, *P*=0.026) (87). In another study, patients with a dPCR < 0.468 experienced a significantly higher TFR rate at 2 years than patients with dPCR ≥ 0.468 (83% vs. 52% *P* = 0.0017). Nevertheless, RT-qPCR was unable to identify patients with a higher risk of relapse after TKI discontinuation (88). In the LAST study, dPCR was administered in patients with undetectable BCR-ABL1 by RQ-PCR. The molecular recurrence rate for patients with detectable BCR-ABL1 by RQ-PCR, for undetectable BCR-ABL1 by RQ-PCR but detectable by dPCR, and for undetectable BCR-ABL1 by both dPCR and RQ-PCR were 50.0%, 64.3%, and 10.3%, respectively (*P*≤ 0.001). Accordingly, detection of BCR-ABL1 by RQ-PCR or dPCR at the time of TKI discontinuation predicted a higher risk of molecular recurrence (37).

## Novel Strategies Targeting LSCs

**Figure 1:**
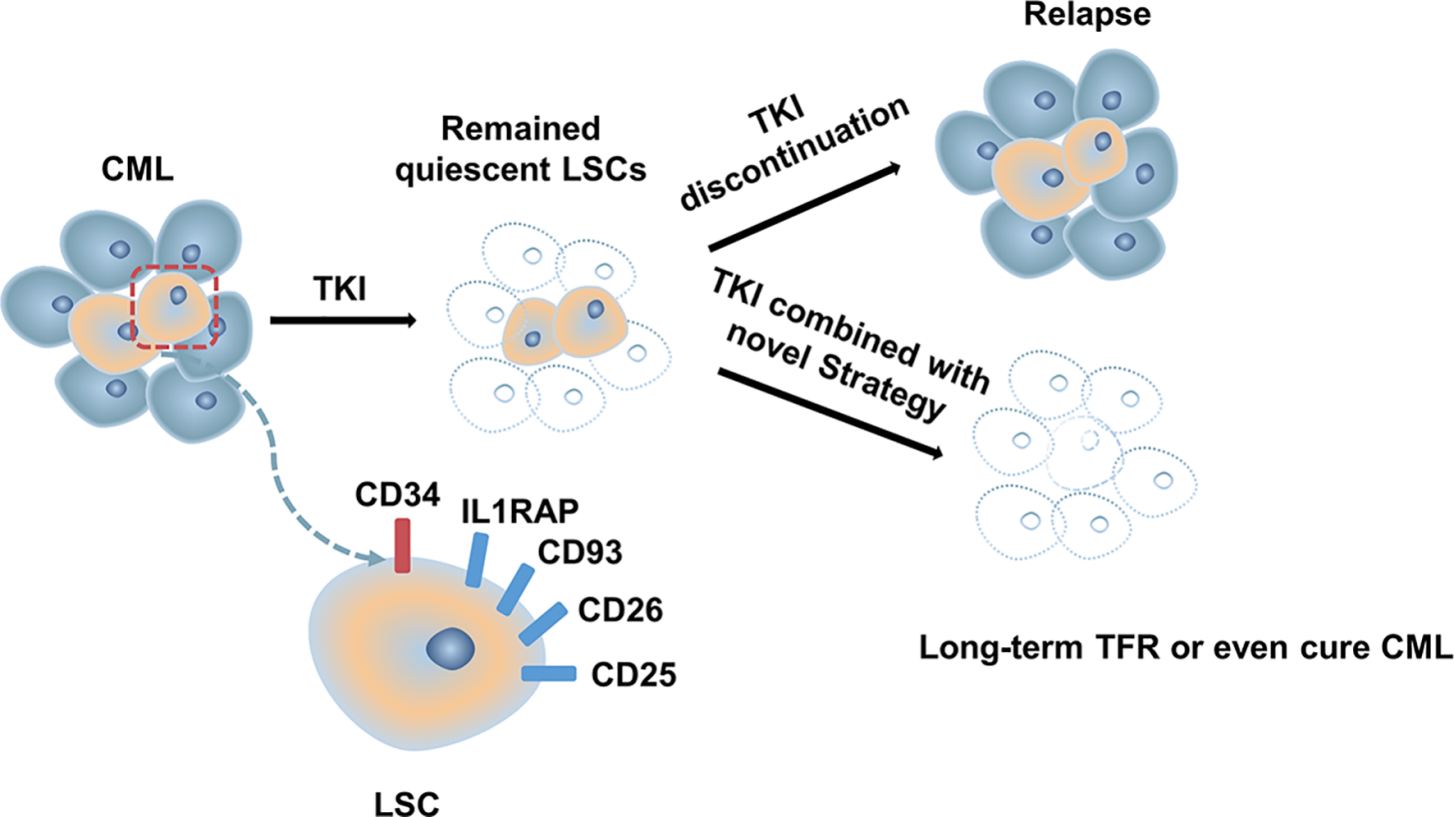
Model for the treatment of CML.

**Figure 2:**
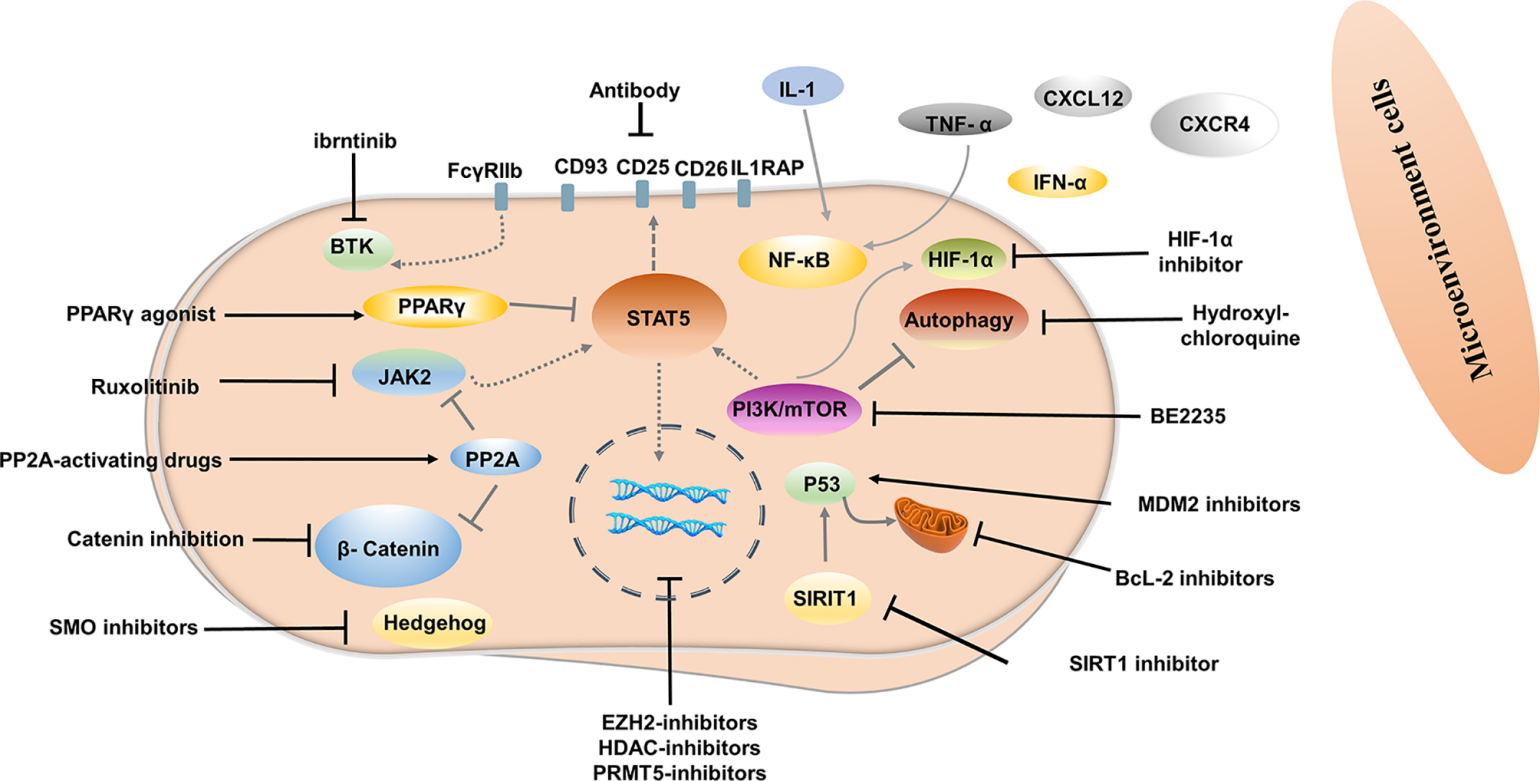
Novel strategies targeting CML leukemia stem cells.

Although most patients with CML-CP achieve a good response with TKI, approximately half of them can successfully discontinue TKI (13, 34, 89). Disease progression and relapse after TKI discontinuation is a conundrum that remains unresolved. Evidence from several studies demonstrated that TKIs act mainly on highly proliferating leukemic cells but have modest effects on CML stem cells (LSCs), which can survive using kinase-independent mechanisms (90, 91). Single-cell RNA sequencing analysis used to distinguish between Bcr-Abl-positive and negative stem cells showed that malignant stem cell populations with quiescence-related genetic features persisted after treatment (92) (**Figure 1**). More emphasis should be placed on kinase-independent mechanisms and targeting quiescent, insensitive LSCs to achieve long-term survival in patients. In recent years, many researchers sought to further study LSCs, and many promising novel strategies targeting LSCs have been developed, aiming to eliminate LSCs and improve outcomes of patients (**Figure 2**).

### Targeting Bone Marrow Microenvironment

Signals from the bone marrow microenvironment play a crucial role in quiescent LSCs escaping TKI therapy (93). The C-X-C motif chemokine ligand 12 (CXCL12) is a major chemoattractant for the homing process and plays a major role in their localization to regulatory niches of LSCs and normal HSCs (94). Disturbed expression of the C-X-C chemokine receptor type 4 (CXCR4) in CML LSCs or CXCL12 targeting in CML LSCs can affect the homing process. Moreover, up-regulation of CXCR4 by TKI contributed to the migration of LSCs to the bone marrow stroma and promoted the survival of quiescent LSCs (95). CXCR4 inhibitor, plerixafor, in conjunction with TKI has also been shown to override drug resistance (96). CXCR4 antagonist BKT140 combined with TKI was found to overcome the protection of bone marrow stroma and reduce the growth of CML cells both *in vitro* and *in vivo* (97). Deletion of CXCL12 from mesenchymal stem cells increased the elimination of LSC by TKI treatment (98). Novel therapy interrupting the CXCR4-CXCL12 axis may weaken the protective role of the bone marrow microenvironment, enhance the sensibility of CML cells to TKI, and even eliminate LSC.

Evidence has shown that autocrine tumor necrosis factor-α (TNF-α) production in LSCs promotes their survival by inducing NFκB/p65 activity, independent of BCR-ABL kinase. TNF-α inhibitor combined with TKI induced significantly higher levels of apoptosis of LSCs compared to either treatment alone, as TNF-α inhibitor showed no off-target inhibition of BCR-ABL kinase activity (99). In another study, TNFα antibody infliximab combined with TKI impaired LSCs growth (100). Moreover, TNF-α signaling was found to mediate expansion and increased expression of CXCL1 in 6C3+ stromal progenitors, and higher expression CXCL1 signaling through CXCR2 enhanced growth capacity and self-renewal of LSCs. Interestingly, CXCR2 inhibitor in combination with TKI remarkably impaired the long-term regenerative capacity of LSCs *in vivo*, with minimal impact on the total peripheral blood count of normal mice (101). IL-1 signaling has also been found to contribute to the overexpression of inflammatory mediators in CML LSCs, indicating that blocking IL-1 signaling can modulate the inflammatory environment. Combined treatment with IL-1RA for IL-1 blockade significantly enhanced LSC elimination compared with TKI alone, which may be associated with additional inhibition of NF-κB signaling (102). Indeed, before the advent of Imatinib, IFNα was used as first-line therapy and was more active against primitive CML progenitor cells than imatinib treatment, which preferentially targeted more mature, differentiated CML progenitor cells (103). In addition, results from *an in vivo* study showed that IFN-α activated dormant stem cells, sensitizing them to the killing effects of subsequent therapeutic agents (104), thus supporting IFN-α combined with a TKI may be a promising strategy to improve outcome. The interim analysis results in phase III clinical study of TIGER (CML V) showed that the upfront accession of Peg-IFN to Nilotinib further increased MR4.0 and MR4.5 rates, which may translate into higher TFR rates (105). In addition, the MRFS rate was significantly higher in patients treated with IFN for a longer period before initiating TKI and in patients using IFN after stopping TKI (106, 107).

### Targeting LSCs *via* Molecular Pathways

Targeting key genes required to regulate LSCs survival but not normal hematopoietic stem cells (HSCs) is an important strategy to inhibit LSCs. Several signal pathways are involved in the regulation of survival and proliferation of LSCs, including Wnt/β-catenin, Hedgehog, MAPK/MNK1/2, mTOR, PTEN, PP2A, Alox5, JAK/STAT, SIRT1, and others (108–115). Inhibitors or agonists targeting some of these signal pathways have been developed and investigated. For example, inhibition of β-catenin by C82, deregulation of the hedgehog by smoothened antagonist LDE225, PP2A-activating drugs, JAK2/STAT5 inhibition ruxolitinib has been shown to reduced survival of LSCs (116–119). A Peroxisome Proliferator-Activated Receptor gamma (PPAR-g) agonist, Pioglitazone is currently used as an antidiabetic agent without hypoglycemic effects in healthy humans. Pioglitazone has been reported to alter the quiescent state of LSCs by reducing STAT5 transcription, thus sensitizing them to TKI (120). In another study, LSCs apoptosis triggered by PPARγ agonist rosiglitazone is related to increased expression of Scd1, Pten, and p53 (121). In the ACTIM phase 2 clinical trial, the cumulative incidence of MR4.5 was 56% in patients who yielded MMR but did not achieve MR4.5 after 12 months of pioglitazone combined with imatinib treatment (122). In a recent study, FcγRIIb was demonstrated to be upregulated in primary CML stem cells, with BTK as a major downstream mediator. Targeting the Bcr-Abl-FcγRIIb-BTK axis by ibrutinib combined with TKI remarkably enhanced apoptosis in quiescent LSCs and thus contributed to eradicating LSCs, suggesting that combining TKI therapy along with BTK inhibition could be a potential approach targeting LSCs (123). Hypoxia-inducible factor-1α (HIF-1α) is a key regulator of the cellular and systemic adaptation to low oxygen (124). In murine models of CML, HIF1α has been documented to have a critical role in the survival and proliferation of LSCs. Deletion of HIF-1α inhibited CML proliferation by impeding cell cycle procession and inducing apoptosis in LSCs (125). *In vivo and in vitro* studies have demonstrated that HIF-1 inhibitors reduce the survival and growth of CML cells and decrease the sustenance of LSCs, but without serious effects on non-CML hematopoietic cells (126).

### Targeting LSCs *via* Bcl-2 and P53 Modulation

The splice variants encoded by BCL2 family genes have pro-and anti-apoptotic functions that contribute to leukemogenesis, CML progression, TKI resistance (127). BCL-2 is overwhelmingly expressed in LSCs and is further increased when patients advance to blast crisis (128). Emerging evidence has shown that combining targeting of BCL-2 by BCL-2/BCL-XL or pan-BCL-2 inhibitors and BCR-ABL tyrosine kinase can enhance the eradication of quiescent LSCs (128–130). Venetoclax, a BCL-2 -selective inhibitor, has shown potent activity in inhibiting the growth of BCL-2-dependent hematological cancers but spares platelets, thus avoiding pronounced thrombocytopenia caused by BCL-XL inhibition (131). Preclinical studies showed significant synergistic effects between venetoclax and TKI on eradicating CD34^+^CD38^−^, CD34^+^CD38^+^, and quiescent stem/progenitor CD34^+^ cells (132). In a recent retrospective study, nine CML-BP patients treated with venetoclax in combination with TKIs experienced an overall response rate of 75% and overall survival of 10.9 months (133).

P53 is crucial in tumor suppression, which can activate the pro-apoptotic BCL-2 family BAX, PUMA, NOXA, and BID and antagonize the anti-apoptotic proteins BCL-2 and BCL-XL triggering apoptosis (134, 135). Targeting p53 in combination with TKI is emerging as a potential strategy to eliminate CML LSCs. SIRT1 is, in fact, an important p53 regulator and is overexpressed in LSCs. As mitochondrial respiration is not affected by TKI treatment, *in vitro* and *in vivo* deletion of SIRT1 inhibited expression of mitochondrial genes and enhanced sensitivity to TKI (136). Inhibiting of SIRT1 or SIRT1 knockdown also increased apoptosis in LSCs and reduced their growth by activating p53 (137). In recent years, activation of p53 by inhibiting MDM2 (the E3 ligase of p53) in combination with TKI has been investigated. The results showed that TKI in combination with MDM2 inhibitor markedly induces apoptosis of LSCs and enhances the efficacy of TKI by inducing pro-apoptotic and suppressing anti-apoptotic Bcl-2 proteins (138–140).

### Targeting Autophagy in LSCs

Autophagy is a lysosomal-mediated, self-degrading process involved in maintaining cellular homeostasis by recycling and decomposing impaired or senescent organelles through the formation of autophagosomes (141, 142). Autophagy may also promote tumor survival by assisting tumor cells to adapt to metabolic stress and evade apoptosis induced by anticancer drugs (143). In CML, autophagy has been documented to be induced in TKI-treated LSCs, which express higher levels than differentiated cells and act as a survival mechanism (144, 145). Several studies have shown that lysosomotropic agent hydroxychloroquine (HCQ) eliminated CML cells and enhanced LSCs to TKI-mediated apoptosis (146, 147). Intriguingly, Lys05, a highly potent lysosomotropic agent, reduced LSCs quiescence and targeted xenografted LSCs combined with TKI treatment (148). Autophagy inhibitors were also among the first compounds to be evaluated in the clinic. The CHOICES study, which explored the safety and efficacy of imatinib (IM) and hydroxychloroquine (HCQ) compared with IM alone, showed MMR rates were 92% and 80% (*P*=0.21) in patients with IM/HCQ and IM at 12 months, while DMR/MMR rates were75% and 66.7% in patients with IM/HCQ and IM at 24 months (149).

### Epigenetic Targeting

Besides the acquisition of genetic lesions, LSCs also exhibit epigenetic dysregulation and reprogramming. Recently, epigenetic therapies have been demonstrated to effectively eliminate LSCs and provide a potential cure for CML in combination with TKI. EZH2, a histone methyltransferase, is a polycomb repressive complex 2 (PRC2) component and is overexpressed in CML LSCs (150). PRC2 is dysregulated in LSCs and coupled with extensive reprogramming of H3K27me3 targets, resulted in altered dependency of the survival in LSCs on EZH2 compared to normal cells (151). In another study, EZH2 inhibitors inhibited colony formation of both human LSC and LSCs with the T315I mutation but spared HSCs. EZH2 deletion also markedly reduced leukemic cells, delayed disease progression, and prolonged survival compared to the control *in vivo* (152). EZH2 inhibitors in combination with TKI led to significantly increased apoptosis and a significant reduction in colony formation in LSCs compared to TKI treatment alone, even in the undivided “TKI-persistent” cells, which may be associated with the reactivation of pro-apoptotic targets and/or promotion of apoptosis downstream of p53 by overcoming BCL6 and EZH2-mediated inhibition of p53 upstream (151).

Histone deacetylase inhibitors (HDACi), a group of promising anticancer agents, can induce apoptosis in nonproliferating cancer cell lines by modulating gene expression through increased histone lysine acetylation (153). HDACi combined with TKI resulted in significantly increased apoptosis of quiescent CML CD34+ cells highly resistant to TKI, which may be associated with downregulation of HOX-, MYC- and WNT-related genes and the reduced expression of E2F-regulated genes (154). In another study, HDACi JSL-1 combined with TKI enhanced the elimination of LSCs and sensitized LSC cells to TKI through γ-catenin-independent mechanisms (155). Chidamide, a novel selective HDACi, markedly reduced the transcript levels of Bcr-Abl and β-catenin and induced apoptosis in LSC when combined with TKI, but exhibited little toxicity towards normal CD34+ progenitor cells (156). Protein Arginine Methyltransferase (PRMT5) was overexpressed in human CML CD34+ cells. Targeting PRMT5 with the small-molecule inhibitor PJ-68 reduced the survival and renewal capacity of LSCs by suppressing the Wnt/β-catenin pathway and increased levels of negative regulators p15^INK4B^ and p27^KIP1^ (157). These findings suggest that epigenetics-based therapies may have a potential role in eradicating LSCs.

### Targeting LSCs *via* Surface Markers

Since LSCs and HSCs express similar cell surface markers, additional markers that can distinguish LSCs from HSCs have been investigated, providing an opportunity to prioritize the use of antibodies against LSCs. IL1RAP was identified as a unique cell surface biomarker distinguishing Ph (+) from Ph (-) LSCs by FISH (158). Targeting IL1RAP by antibodies and novel CAR T-cell therapy has been found to exert anti-leukemic effects *in vivo and in vitro* through specific killing, with no severe negative effects on normal HSC (159, 160). CD26 (DPPV) has also proved to be a novel, specific biomarker for CML LSCs, which is promising for the diagnosis and targeted treatment of CML (161–163). In a recent study, a venetoclax-loaded immunoliposome remarkably induced apoptosis in CD26+ cells in both stem cells and progenitor cells population (164). However, DPPIV blocker vildagliptin in combination with nilotinib did not exert a synergy effect, indicating insignificant effects of co-administration (165). CD25 is a novel STAT5-dependent marker of LSCs, whose expression is upregulated by the PI3K/mTOR blocker BEZ235. In addition, BEZ235 produced synergistic antitumor effects on CML cells when combined with nilotinib or ponatinib (166). Recently, CD93 has also been identified as a novel marker on LSC and persisted in patients with molecular recurrence after TKI discontinuation (167, 168). CD25 and, CD93 are potentially promising targets for LSCs-eradicating immunotherapies. These significant findings support the approach of targeting surface antigens of LSCs for LSCs elimination.

Targeted agents targeting the above molecules or pathways have shown promising efficacy in eliminating LSCs by enhancing the killing effect of TKI on LSCs *in vitro* and *vivo* studies. Several promising strategies have also entered clinical trials, and some preliminary results showed that TKIs in combination with IFN-α, JAK2 inhibitors, PPAR-γ agonists, BCL-2 inhibitors, and lysosomotropic agents have the potential to improve treatment response in CML (169, 170).

## Conclusions

In clinical studies and the real world, some patients who achieve a stable DMR can successfully discontinue first- or second-generation TKI. However, caution should be taken in MMR patients attempting TFR outside of clinical trials. There remains a possibility for patients who fail a first TFR to discontinue TKI with close monitoring. Patients with a deeper molecular response and longer molecular response duration before stop TKI have decreased risk of molecular relapse. Some immunological indicators such as NK cell counts and NKG2D can also contribute to identifying patients suitable for TKI discontinuation. Developing models that incorporate relevant predictors to predict the likelihood of maintaining TFR is clinically important. Since TKI de-escalation may alter the immune response against leukemia and preserve the long-term efficacy of standard dose TKI therapy while reduce adverse events, the de-escalation approach to TFR may be a promising strategy aimed to improve TFR. New molecular monitoring techniques and novel strategies contributing to the eradication of LSCs are currently under evaluation and are expected to yield preferable outcomes for improving TFR. Importantly, there is hope to expand the TFR population and improve TFR in clinical practice shortly.

## Author Contributions

YC, JZ, FC, and WL contributed to manuscript revision, read, and approved the submitted version.

## Conflict of Interest

The authors declare that the research was conducted in the absence of any commercial or financial relationships that could be construed as a potential conflict of interest.

## Publisher’s Note

All claims expressed in this article are solely those of the authors and do not necessarily represent those of their affiliated organizations, or those of the publisher, the editors and the reviewers. Any product that may be evaluated in this article, or claim that may be made by its manufacturer, is not guaranteed or endorsed by the publisher.
